# A review of HIV pre‐exposure prophylaxis (PrEP) programmes by delivery models in the Asia‐Pacific through the healthcare accessibility framework

**DOI:** 10.1002/jia2.25531

**Published:** 2020-06-30

**Authors:** Janice YC Lau, Chi‐Tim Hung, Shui‐Shan Lee

**Affiliations:** ^1^ Stanley Ho Centre for Emerging Infectious Diseases The Chinese University of Hong Kong Hong Kong People’s Republic of China; ^2^ JC School of Public Health and Primary Care The Chinese University of Hong Kong Hong Kong People’s Republic of China

**Keywords:** pre‐exposure prophylaxis, HIV prevention, implementation, delivery model, accessibility, Asia‐Pacific

## Abstract

**Introduction:**

In the Asia‐Pacific, pre‐exposure prophylaxis (PrEP) is a newly introduced public health intervention for minimizing HIV transmission, the coverage of which has remained limited. The best delivery models and strategies for broadening access of the vulnerable communities are not fully known. This review identified PrEP programmes reported in the Asia‐Pacific, which were classified by delivery models and assessed with a healthcare accessibility framework.

**Methods:**

We performed a literature search on PubMed and Ovid MEDLINE using relevant search terms, manual searched grey literature by visiting relevant websites, examined reference lists and contacted authors for clarification of included PrEP programmes reported through July 2019. A structured table was used for data extraction and summarizing findings in accordance with the five constructs of approachability, acceptability, availability, affordability and appropriateness grounded in the conceptual framework of Healthcare Accessibility.

**Results and discussion:**

This literature search yielded a total of 1308 publications; 119 full texts and abstracts were screened, and 24 publications were included in the review. We identified 11 programmes implemented in seven cities/countries in the Asia‐Pacific. A typology of four PrEP delivery models was delineated: (a) fee‐based public service model; (b) fee‐based community setting model; (c) free public service model; and (d) free community setting model. Overall, the free community setting model was most commonly adopted in the Asia‐Pacific, with the strength to boost the capacity of facility and human resources, which enhanced “approachability”, “availability” and “acceptability.” The free public service model was characterized by components designed in improving “approachability,” “availability” and “appropriateness,” with attention on equity in accessing PrEP. Among free‐based models, long‐term affordability both to the government and PrEP users would need to be maximized to increase accessibility. Alongside the need for raising awareness, supportive environments and ensuring timely access were means for enabling the development of a sustainable PrEP service.

**Conclusion:**

PrEP programmes could be classified by delivery models through the five constructs of healthcare accessibility. While the coverage of PrEP remains limited in the Asia‐Pacific, an evaluation of these models could benchmark best practices, which would in turn allow effective models to be designed.

## 1 Introduction

Pre‐exposure prophylaxis (PrEP) with coformulated tenofovir/emtricitabine is an intervention for HIV prevention, which could, with high adherence achieve an effectiveness of >90%, as evidenced in many clinical trials [1, 2, 3, 4]. In 2015, a policy brief was released by the World Health Organization (WHO), recommending PrEP as an additional option for offering to those at substantial risk of HIV acquisition and as part of the HIV prevention strategy planning [5]. However, utilization of PrEP has remained low [6, 7]. Barriers leading to its low utilization include poor awareness [8], perceived side‐effects [9, 10, 11], concerns about maintaining adherence [8, 9], costs [11, 12, 13] and anticipated stigma [11, 14, 15, 16]. At‐risk populations including men who have sex with men (MSM) in India [9], transgender women (TGW) in the United States (US) [10], and female sex workers (FSW) in Zimbabwe [16] reported difficulties to access the antiretrovirals for PrEP.

From fledgling pilots to implementation studies, various models of PrEP delivery aiming to optimize its benefits have evolved worldwide, ranging from the pharmacy‐based model in the US [17], nurse‐led model in Canada [18], and community‐ and facility‐based model in Kenya [19]. In the Asia‐Pacific, the estimated HIV prevalence was high at 5.9 million in 2018. The epidemic has concentrated in people who inject drugs (PWID), MSM and FSW, causing major health and economic consequences [20]. While PrEP has been introduced to reduce the vulnerability to HIV infection in some places, the best delivery models and factors leading to their success, are not fully known. In this context, what constitute easy and broad access of PrEP is important for the development of a scalable model. In the past decades, conceptual frameworks defining access as determined by population and system characteristics [21], supply and demand factors [22], and ability to obtain care [23] were commonly used in health service research. The Levesque framework refined previous ones by specifying five key constructs: approachability, acceptability, availability, affordability and appropriateness, and with five corresponding abilities attributed to service users: ability to perceive, to seek, to reach, to pay and to engage [24]. This framework has been applied in a range of healthcare contexts for analysis [25, 26, 27]. In this study, we set out to identify PrEP delivery models in the Asia‐Pacific, and chose the Levesque framework to assess their strengths and weaknesses through the five constructs to understand healthcare access from multiple levels.

## 2 Methods

A literature search was conducted to identify published evidence that have evaluated the outcomes of PrEP‐related projects in the Asia‐Pacific. We examined the publications by searching the following electronic bibliographic databases in March 2019: PubMed, and Ovid MEDLINE, using the following search terms: “(pre exposure prophylaxis OR prep) AND (service OR model OR programme *' OR pilot OR package OR project OR intervention OR care adj3 model OR trial OR rollout OR scale up OR evaluate *' OR implemented *' OR delivery *') AND (risk behaviour OR adherence OR condom OR access OR awareness) AND HIV”. Limit was set to English language publications and human studies and date from 1 January 2010. The identified articles were screened by title, abstract and then the full text. We completed manual search in April 2019 by visiting websites of key organizations involved in HIV prevention and PrEP‐related studies or services (e.g. Global Advocacy for HIV prevention (AVAC), PrEP Watch, PrEP Fact, PrEP MAP) for PrEP‐related information. We extended our search by examining reference lists cited in the selected publications, and reviewing on the latest evidence reported in the International AIDS Society (IAS) in July 2020.

In this study, “PrEP delivery” is defined as the implementation of the provision of PrEP for HIV prevention in the real‐world setting, including its operation as a standalone service or as part of an integrated HIV prevention programme. To be included for review, a study should have: (i) examined PrEP service components and outcomes; (ii) presented types of evidence in quantitative, qualitative or mix‐method approach, and (iii) been published as a peer‐reviewed journal article, or conference presentations. When one study had led to multiple reports showing apparent duplications, we reviewed the principal paper (with the greatest number of subjects). A study with multiple reports involving multiple study sites has also been included. For the purpose of retrieving the most up‐to‐date research findings for the review, we have also included conference papers and abstracts reporting the design and interim results of ongoing projects or those conducted in phases, in addition to peer‐reviewed articles. We have subsequently emailed authors of included studies for clarification of information reported, and confirmed if there are additional relevant publications that might have been published or become publicly accessible. We excluded publications that were review pieces, opinions, letters, dissertations, commentaries or editorials.

Publications meeting the inclusion criteria were reviewed for the identification of PrEP programmes. For each identified programme, we extracted data on populations, location and access points. A structured table was used for summarizing findings regarding the five constructs of approachability, acceptability, availability, affordability and appropriateness [24]. Data synthesis and presentation was supported by the application of spider chart, with five axes showing the value of each parameters. The value of each parameter was scored according to the operational inputs, for example, preparation of actions, technical or logistical strategies, and enabling conditions in broader context (e.g. funding) that support PrEP accessibility. The identified models were rated by the following 5‐point scoring scale: 1 very low, 2 low, 3 moderate, 4 high and 5 very high. The score was a reflection of the degree of availability of the types of services meeting the needs of the clients served. A higher score was given to a service with more abundant operational inputs as reported. The authors independently scored the models, the results of which were then combined and averaged to give the final scores. Outcomes relevant to accessibility (e.g. beneficiaries, providers’ capacities or users’ experience) were also included for evaluation.

## 3 Results and Discussion

**Figure 1:**
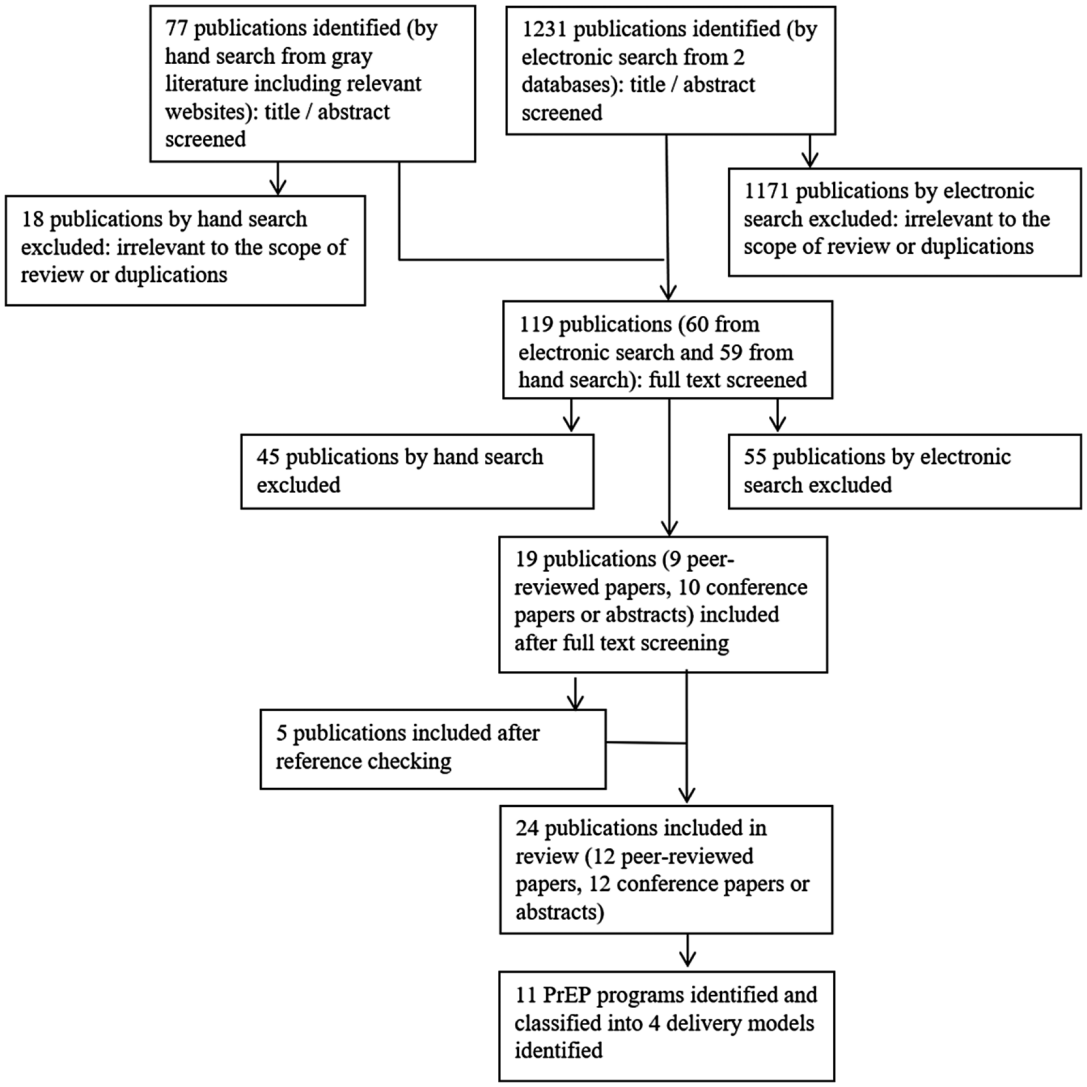
Flow chart of identification of PrEP delivery models in the Asia‐Pacific.

This literature search yielded a total of 1308 publications, 1231 of which were identified through electronic search (787 from PubMed and 444 from Ovid MEDLINE), plus a further 77 by manual search from the grey literature. From 119 full texts and abstracts screened, and five additional items from reference list checking, 24 publications were identified, and the contents synthesized. Figure 1 presents a flow diagram of the article screening process and breakdown of sources that have contributed to the review. Eleven programmes in Thailand [28, 29, 30, 31, 32, 33, 34, 35, 36], Vietnam [35, 37], India [38, 39, 40], Australia [41, 42, 43, 44], New Zealand [45, 46, 47, 48], Taiwan [49, 50] and Philippines [51] were identified with a brief description of each displayed in Table 1. Classification of the 11 identified programmes results in a typology of four PrEP delivery models: (a) fee‐based public service model; (b) fee‐based community setting model; (c) free public service model; and (d) free community setting model (Table 2). The typology distinguishes two core dimensions of PrEP services in the 11 programmes: financing that divides free‐of‐charge versus out‐of‐pocket payment required for the services, and access points that divides public hospital/clinics (services) versus community settings, which involved a range of settings including private clinics and those operated by community‐based organization (CBO).

**Table 1:** Brief descriptions of PrEP programmes available in the Asia‐Pacific

Programme name	Year	Location	Populations	Access point(s)	References
PrEP‐30	Dec 2014	Thailand	Males & females	TRCARC^a^ Anonymous clinic	Colby et al. [28]
					Colby et al. [29]
Test, Treat, and Prevent HIV programme	April 2015	Thailand	MSM, TGW	5 hospitals‐based outpatient clinics in Thailand, with 2 delivered PrEP: (i) Lerdsin Hospital in central Bangkok; (ii) Thammasat Hospital in Prathum Thani	Ongwandee et al. [30]
PrEP substudy	Oct 2015	Thailand	MSM, TGW	4 Thai urban community clinics: RSAT^b^ and SWING^c^ in Bangkok; SWING^c^ & Sisters^d^ in Pattaya	Plotzker et al. [31]
O2O Programme	Jan 2016	Thailand	Male at birth	4 clinics in Bangkok (TRCARC^a^ Anonymous Clinic, Adam’s Love Clinic, & 2 community‐based drop‐in centres operated by RSAT^b^, & SWING^c^)	Anand et al. [32]
					Anand et al. [33]
Princess PrEP programme	Jan 2016	Thailand	MSM, TGW	8 community health centres operated by CBOs in Chiang Mai, Bangkok, Chonburi & Songkhla	Phanuphak et al. [34]
					Ramautarsing [35]
					Seekaew et al. [36]
KP‐led Prepped for PrEP‐P4P	Mar 2016	Vietnam	MSM, TGW, FSW, PWID & their sex partners	11 CBOs (assessment); 4 private clinics & 5 public clinics (PrEP prescription) in Ho Chi Minh & Hanoi	Ramautarsing [35]
					Green [37]
PrEP‐India	2016	India	FSW	Ashodaya Samithi clinic in Mysore, Karnataka, India; DMSC^e^ Clinic in Kolkata, West Bengal, India	Reza‐Paul [38]
					Reza‐Paul et al. [39]
					Reza‐Paul [40]
EPIC‐NSW	Dec 2016	Australia	MSM, TGW, & high‐risk heterosexual men & women	10 public sexual health clinics in New South Wales	Schmidt et al. [41]
					Zablotska et al. [42]
					Grulich et al. [43]
					Vaccher et al. [44]
NZPrEP	Feb 2017	New Zealand	GBM	4 publicly funded sexual health clinics in Auckland; 2 pharmacies (PrEP dispensation)	Saxton et al. [45]
					Myers et al. [46]
					Saxton et al. [47]
					Azariah et al. [48]
PrEP‐PAPA Project	Aug 2017	Taiwan	High risk individuals	1 public hospital in Taiwan (assessment)	Ku [49]
					Chu et al. [50]
Project PrEPPY	2017	Philippines	MSM, TGW	2 Love Yourself’s clinic in Manila	Rosadiño [51]

^a^ Thai Red Cross AIDS Research Centre.

^b^ Rainbow Sky Association of Thailand.

^c^ Service Workers IN Group.

^d^ Sisters Foundation.

^e^ Durbar Mahila Samanwaya Committee.

**Table 2:** Classification of 11 PrEP programmes into four delivery models

PrEP delivery models	PrEP programmes
(a) Fee‐based public service model	KP‐led Prepped for PrEP‐P4P
	PrEP‐PAPA Project
(b) Fee‐based community setting model	PrEP‐30
	KP‐led Prepped for PrEP‐P4P
(c) Free public service model	Test, Treat, and Prevent HIV programme
	EPIC‐NSW
	NZPrEP
(d) Free community setting model	PrEP substudy
	O2O Programme
	Princess PrEP programme
	PrEP‐India
	Project PrEPPY

**Figure 2:**
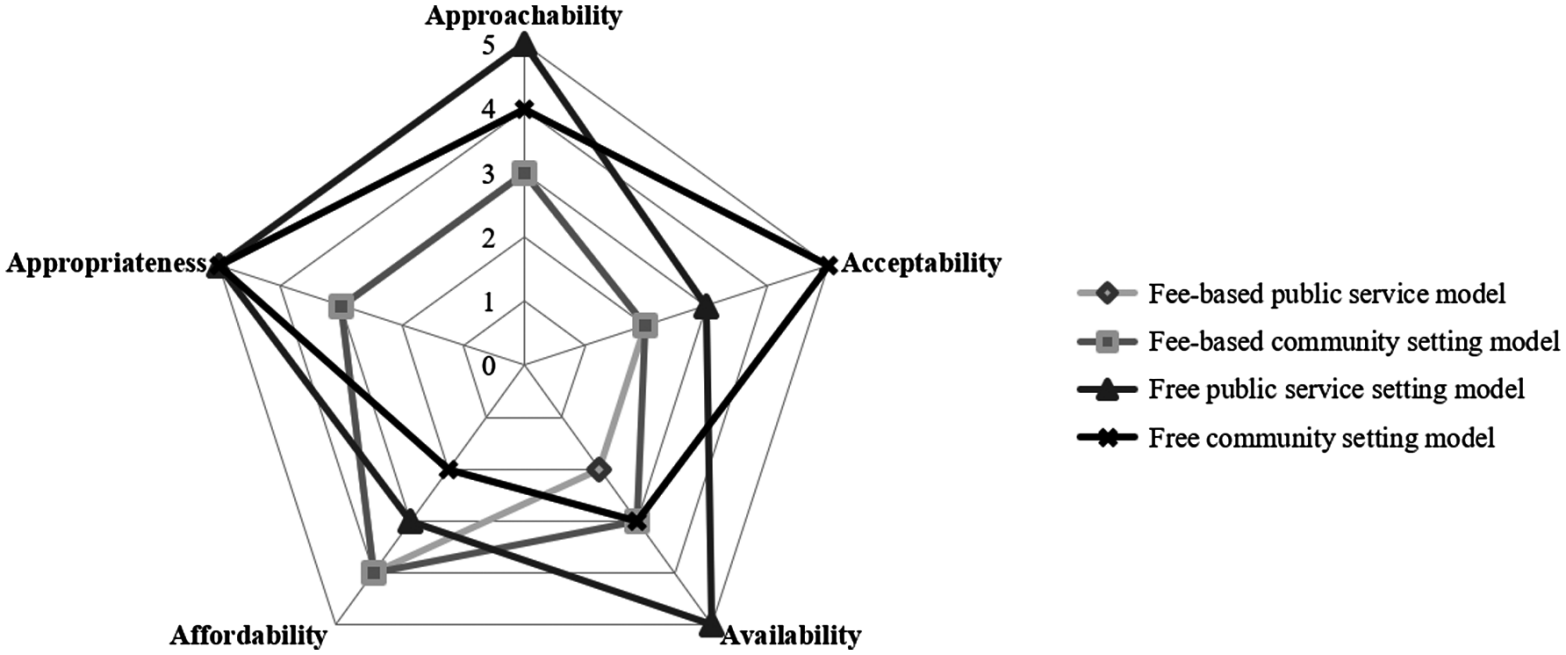
Spider chart of PrEP delivery models in the Asia‐Pacific.

**Figure 3:**
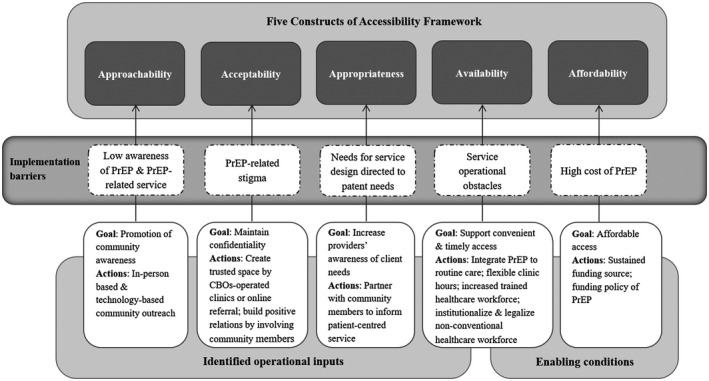
Modified conceptual framework of Healthcare Accessibility for PrEP delivery adapted from Levesque et al.

Each reviewed programme may fit into more than one model depending on how the bundle of services falls into the variables for classification. For the KP‐led Prepped for PrEP‐P4P, it was classified under both the fee‐based public service model, and fee‐based community setting model on the basis of the availability of its assessment service in the community (i.e. CBOs), and PrEP prescription in both community (i.e. private clinics) and public setting (i.e. public clinics). While each model has distinct characteristics in programme design, it often took on a blend of strategies. An overview of strategies (Table 3), and the strength and weakness of these models (Figure 2) with respect to the five constructs of Healthcare Accessibility are elaborated in the following results. In analysing the results from this review, the Levesque framework was modified with determinants grouped under “identified operational inputs” and “enabling conditions” to address implementation barriers impeding the achievement of accessibility (Figure 3).

**Table 3:** Overview of strategies for examining PrEP programmes within the five constructs of the Healthcare Accessibility framework

Model	Programme name	Populations	Approachability	Acceptability	Availability	Affordability	Appropriateness
Fee‐based public service model	*KP‐led Prepped for PrEP‐P4P*	MSM, TGW, FSW, PWID & their sex partners	Educational materials Social media promotion campaigns	Partnership with CBOs & KP‐led social enterprises Confidential online portal by Toi Hen (‘I Reserve’) mobile app	Integration of PrEP service to public sector Training support for providers Referral of eligible participants from CBOs to clinics	Affordable low‐cost fee at US15 per month for generic PrEP Ongoing project	Service needs assessment Community engagement to co‐create service model HIV testing PrEP adherence support by f2f interaction
	*PrEP‐PAPA Project*	High risk individuals	Educational materials	[Lack of details]	Training support for healthcare providers: PrEP workshops & online PrEP tutorial	Affordable low‐cost fee at US37.3 (NTD 1150) per month for generic PrEP [Lack of details about project status]	Laboratory tests in Taiwan Continuous clinical follow up, monitoring and counselling in Taiwan
Fee‐based community setting model	*PrEP‐30* *	Males & females	[Lack of details]	[Lack of details]	Integration of PrEP service to routine service	Affordable low cost at <US$1 (30 THB) per day Ongoing project	HIV testing Provision of PrEP at first visit Counselling at follow up visits
Free public service model	*KP‐led Prepped for PrEP‐P4P*	MSM, TGW, FSW, PWID & their sex partners	Educational materials Social media promotion campaigns	Partnership with CBOs & KP‐led social enterprises Confidential online portal by Toi Hen (‘I Reserve’) mobile app	Integration of PrEP service to public sector Training support for providers Referral of eligible participants from CBOs to clinics	Affordable low‐cost fee at US15 per month Ongoing project	Service needs assessment Community engagement to co‐create service model HIV testing PrEP adherence support by f2f interaction
	*Test, Treat, and Prevent HIV programme*	MSM, TGW	Educational materials In‐person‐based community outreach by peer‐driven recruitment intervention	Trained staff at sites explaining benefits of PrEP to walked‐in individuals	One‐stop HIV service Training support for peer‐recruiters	Free Completed project	Rapid HIV testing Clients with HIV‐positive results were offered ART Adherence counselling at follow up visits
	*EPIC‐NSW*	MSM, TGW, & high‐risk heterosexual men & women	Educational materials Onsite and outreach recruitment by peer educators	Peer educators from ACON^a^ delivered information at follow‐up visits Peer educators administered risk assessment	Efficient and cost‐effective care by task‐shifting Training support for nurses Training support for peer educators After hours at CBOs‐testing sites Telephone triage and walk‐in service to support urgent access	Free Ongoing project	Rapid HIV testing PrEP prescription on first day appointment Medical follow‐up for clients with abnormal results (e.g., HIV positive) Multidisciplinary follow‐up for clients with chronic viral hepatitis or other issues Adherence support in follow up visits Clients’ autonomy to continue, stop and restart PrEP was respected
	*NZPrEP*	GBM	Educational materials Ethnicity quotas for non‐European & indigenous Maori Social media promotion Educational forums were hosted in 4 cities “Map of doctors” mobile app	Peer educators from NZAF^b^ & BP^c^ provided risk reduction counselling	Training support for healthcare providers Collaborative teamwork to develop patient consent form to overcome medicolegal risks PrEP dispensed at 2 participating community pharmacies	Free Ongoing project	2 community NGOs were involved in the study design HIV enzyme immunoassay (EIA) fourth‐generation Medical follow‐up for clients with adverse events Management according to Australasian Society for HIV Medicine guidelines for clients acquired HIV during the study Risk reduction counselling at follow up visits
Free community setting model	*PrEP substudy*	MSM, TGW	Educational materials Social media promotion campaigns Community outreach	Partnership with CBOs: RSAT^d^, SWING^e^, & Sisters^f^ Trained project staff at sites explaining benefits of PrEP to eligible participants	Staff have been trained and were made available at all clinic sites who openly discussed study‐related questions prior to clients’ participation	Free Completed project	HIV testing using 3rd generation rapid antibody test & shipped for laboratory‐based 4th generation assay Referral for clinical care for those with recent acute HIV infection symptoms
	*O2O Programme*	Male at birth	Educational materials Social media promotion campaigns Electronic‐referral based system	Confidential online booking Real‐time e‐Counselling by trained counsellor from Adam’s Love	Free booking & counselling online Same‐day responses by e‐Counselling Prompt electronic notification of booking details	Free Completed project	Clients were free to choose sites of clinical services according to their preference
	*Princess PrEP programme*	MSM, TGW	Educational materials Social media promotion campaigns In‐person‐based community outreach by enhanced peer mobilization (EPM) approach	Partnership with CBOs: CAREMAT, MPlus^g^, SWING^e^, Adam’s Love, RSAT^d^, & Sisters^f^	Flexible clinic visits hours CHW as the resources Training support, & assessment requirement (by practicum & examination) for CHW	Free 3‐year funding support	Community engagement in service design & delivery Third‐generation HIV rapid diagnostic testing Same‐day PrEP Clients’ decision to use PrEP was respected Offered ART for clients with HIV‐positive results Referral of clients with HBsAG positive result to hepatologists or internists PrEP adherence support by life‐steps approach
	*PrEP‐India*	FSW	Educational materials In‐person‐based community outreach	Partnership with CBOs: DMSC^h^ (brothel‐based); Ashodaya (street‐based) Enrolled community leaders to as first PrEP users	Community members as the resources Training support for community members	Free Completed project	Community engagement in service design delivery HIV testing Individualized PrEP dispensation plan Adherence support at clinics
	*Project PrEPPY*	MSM, TGW	Educational materials: culturally sensitive & sex‐positive designed	Partnership with CBO: Love Yourself Foundation	[Lack of details]	Free Completed; still providing PrEP	HIV Rapid Test Counselling at follow up visits

^a^ AIDS Council of New South Wales.

^b^ New Zealand AIDS Foundation.

^c^ Body Positive.

^d^ Rainbow Sky Association of Thailand.

^e^ Service Workers IN Group.

^f^ Sisters Foundation.

^g^ MPlus Foundation.

^h^ Durbar Mahila Samanwaya Committee.

* Project name was changed in June 2018 to PrEP‐15 due to the availability of another generic PrEP drug at lower cost.

### 3.1 Construct 1: approachability

Approachability is defined as the extent that populations “… facing health needs can actually identify that some form of services exists, can be reached, and have an impact on the health of the individual” [24], which could be contributed by health information and outreach efforts. As regards PrEP utilization, a high level of awareness is important in increasing the willingness of potential PrEP users to access the required antiretrovirals to minimize HIV transmission [52]. However, awareness and the use of PrEP have remained relatively low in the Asia‐Pacific, as shown in surveys that 93% MSM and TGW in India were unaware of PrEP [53]; only about 15.1% FSW in Guangxi of China [54], and 5% gay men, other MSM and TGW in Myanmar [55] had knowledge of PrEP. Promotion of PrEP awareness was highlighted in all four models, with varying strategies adopted by individual programme. All models involved disseminating educational materials (both paper and electronic versions) in real‐world (e.g. study sites, local NGOs), or virtual settings (e.g. MSM‐and‐TGW popular websites and apps) to accelerate the reach for targets. In addition, the free community setting model implemented in India (PrEP‐India that targeted FSW) simplified promotional materials in a comprehensible language to broaden the health promotional efforts [39]. To eliminate ethnic disparities in PrEP access as a result of limited knowledge, the free public service model implemented in multicultural societies, such as Australia (EPIC‐NSW) had promotional material available in six most common languages (i.e. Chinese, Thai, Spanish, Indonesian, Portuguese and Arabic), addressing individual‐level barrier arising from linguistic differences [42]. The NZPrEP implemented in New Zealand set equity quotas to ensure equal participation by ethnic minorities, including indigenous Maori gay and bisexual men (GBM) [45, 48].

Beyond awareness, a key strategy to promote PrEP service is to reach key affected populations who are often hidden and invisible resulting from social marginalization. Techniques like in‐person‐based community outreach by trained mobilizers were applied in both the free public service model, and free community setting model. For example, the potential of peer‐driven recruitment intervention to reach HIV‐negative MSM and transgender women was demonstrated in the Test, Treat and Prevent HIV Programme, allowing service providers to take steps to deliver timely HIV prevention services [30]. To solicit the interest of the at‐risk communities, similar strategies were observed in the PrEP‐India [38, 39, 40], and the Enhanced Peer Mobilization (EPM) in the Princess PrEP programme in Thailand [34]. Rather than applying conventional in‐person‐based technique, technology was used by these two models, to achieve similar results. For example, the O2O (Online‐to‐Offline) Programme adopted a unique electronic‐referral‐based system to optimize reach to at risk yet hard‐to‐reach populations. Interested individuals could undergo risk assessment via real‐time e‐Counselling, make online booking and be transited to offline PrEP services including HIV testing [32, 33]. To assist clients to locate PrEP providers, the “map of doctors” mobile application was introduced by NZPrEP to strengthen an individual’s ability to navigate PrEP prescription and link potential users to PrEP service in an easy and timely manner [46]. Whether certain techniques are more effective than the others are unknown, but increasing community awareness and community outreach that contribute to approachability is seemingly one of the priorities of these models.

### 3.2 Construct 2: acceptability

Accessibility consists of the degree of acceptability of services that is determined by “cultural and social factors” [24]. For HIV prevention, social stigma has continued to be the major barrier for accessing effective services. PrEP‐related stigma, such as the label of promiscuity attached to PrEP users, has been reported to be the societal‐level barrier for PrEP access [11, 14, 15, 16]. In the Asia‐Pacific, community involvement was a common component in all models, but the pattern varied regarding what was done, and how outcomes were achieved to address service needs. For example, the free community setting model highlighted the importance of building partnership with CBOs to increase acceptability of individuals in the target populations. CBO‐operated clinics served as important PrEP access points (Table 1), with the advantages of creating and endeavouring an inclusive, non‐judgmental and welcoming space for the clients. Such strategy has the potential to encourage PrEP uptake among those concerned about inviting social discredit for disclosing PrEP use. Study suggested a positive association between the role of CBOs and PrEP adherence. The Princess PrEP Programme had successfully reached the targeted populations, with 1,467 MSM and 230 TGW having started PrEP within 2 years. Self‐reported adherence (at least four PrEP pills per week) of over 90% was achieved over the ensuing year though the retention fell [34]. The same programme reported that 37.4% of participants had low adherence, which was associated with younger age, being a TGW and first‐time clients [36].

Services developed after taking reference of local opinions could have been more adequate of meeting the unique needs of the community. A focus on acceptability of services by involving community leaders and members was shown in all models, though at varying levels of penetration. A higher level of involvement was observed in the free community setting model. The Princess PrEP Programme was, for example, exemplified by the wide participation of dedicated community members who were recruited and trained to become community health workers (CHW) in participating in service provision. Likewise, sustained engagement of FSW was advocated as the ethos of the PrEP‐India project. To foster trust and to promote acceptability of PrEP within the FSW community, FSW leaders enrolled themselves as the first PrEP users. Several strategies, such as active participation in awareness campaign, recruitment, PrEP dispensation and data collection regarding risk behaviours, have been developed to mobilize the community, which entailed the sense of ownership [40]. Targeting to enrol 2,000 FSW, it had successfully recruited a total of 1,325 with good retention [38]. It was reported that the capacity and experience of PrEP‐India project have an impact to “shape the understanding of risk and prevention program” and create “trusted space” among the FSW [39]. The strategy of involving community leaders to increase acceptability of PrEP service could have positively affected the rollout of PrEP [38, 39, 40]. Nevertheless, the lack of faith in the capacity of the FSW community and its commitment for the community‐led process had remained a main challenge in the implementation of this project [40].

Other programmes falling under the free community setting model shared the commonality of underscoring the key role of CBOs and community members. In Philippines, community members volunteering in Project PrEPPY were trained to serve as PrEP navigators and life coaches to address biomedical and psychosocial issues that clients faced [51]. Efforts of this programme have drawn 3,938 participants expressing interest to take PrEP, with 250 started PrEP. However, strategies to address challenge of protecting confidentiality of PrEP users were still needed. In Vietnam, the KP‐led Prepped for PrEP‐P4P provided PrEP at four private clinics and five public hospitals, in combination with 8 KP‐led CBOs with a track‐record of providing highly skilled HIV services. Putting clients at the centre of care by meeting their needs was highlighted by the efforts of needs assessment as well as service co‐creation. The cumulative uptake climbed from 42 to 1,967 in 20 months, with a continuation rate of 75.6% [35]. Such service model had met the preference of users who accessed PrEP through CBOs (71.4%) [37]. PrEP uptake among TGW communities, however, remained relatively low [56].

In Thailand, the O2O Programme had previously demonstrated high effectiveness in other biomedical services [57]. It created significant impacts in providing new opportunities to facilitate stigma‐free access to PrEP. Of the 272,568 people reached online in 2016, 425 engaged in e‐Counselling and 325 received HIV testing; 168 (out of 316 HIV negative) subsequently started PrEP [32]. The same model implemented between May 2017 and August 2018 continued to record high e‐Counselling and PrEP uptake rates [33]. Apparently, these e‐counsellors had fostered positive and supportive relationships with and between clients, facilitating their access in a “virtual non‐judgmental setting.” Protection of confidentiality was largely achieved by the operation of an online booking process requiring the least personal information (i.e. preferred pseudonym and email address), which was a successful attempt to tackle stigma. Similar strategy using confidential online portal was adopted by the KP‐led Prepped for PrEP‐P4P, the outcomes of which were unavailable yet.

### 3.3 Construct 3: availability

The conceptual framework by Levesque et al. specifies that availability, meaning services that “… can be reached both physically and in a timely manner,” influences accessibility of health service [24]. Availability characteristics include the number and type of facilities (e.g. public hospitals, CBOs‐operated clinics, private clinics), scheduling (specific time throughout the day, e.g. daytime hours, evening hours, weekdays, weekends), and healthcare workforce (presence of trained and competent service providers, e.g. physicians, nurses or lay providers). In regard to PrEP service delivery, inadequate healthcare facilities could contribute to operational obstacles to timely access. To tackle infrastructural issues, PrEP services provided at community settings, for example, PrEP‐30, had integrated PrEP services into existing VCT/ART clinic to assist clients’ enrolment, which facilitated a quick start‐up of the programme using existing facilities and workforce. The accumulated number of participants was 197 by December 2015 [28], rising to approximately 2,000 by June 2018 [29]. For the Test, Treat and Prevent Programme provided at the public setting, higher PrEP uptake was observed at sites established for one‐stop HIV service [30]. Offering flexible clinic appointments was a means to minimize clinic visit burden. For example, the Princess PrEP Programme facilitated participation by opening at flexible clinic visit hours to suit the users’ needs [35]. However, meeting the service needs of highly mobile populations could be a challenge. In the same programme, lower adherence was observed in the Chonburi province where a majority of participants were TGW who also engaged in sex work. Factors influencing adherence level were high mobility of sex workers and the irregular time in meeting with clients [36]. Technology can have a practical impact on increasing the availability of services. Technology utilization of the O2O Programme contributed to PrEP access by providing services at any time and any space. Same‐day responses by e‐Counselling and immediate electronic notification of booking details via email facilitated easy access, at a median (IQR) interval of 3 (0 to 7) days between receiving e‐ticket and check‐in.

An adequately sized healthcare workforce plays a critical role in broadening PrEP access. Notably, the impact of the nurse‐led approach in EPIC‐NSW in tackling staffing issues in the process of accelerating a rapid expansion of PrEP access was reported [41]. Authorized by the NSW Ministry of Health, the tasks previously undertaken by doctors were performed by trained registered nurses. Nurses were supported by doctors who regularly reviewed patients’ records and followed up patients with abnormal results. Facilitator of this approach was task‐shifting, allowing an efficient use of human resources, with the potential to improve job satisfaction of both nurses and doctors [41]. The workforce capacity was successfully expanded to support rapid access of up to 3,700 participants in an 8‐month period at the participating sexual health clinics, leading to a rapid decline in HIV infection and HIV diagnosis [43]. Likewise, provider shortage may impact PrEP access. Training provision was included as one of the components across all models, but only a few programmes under the free public service model had specified the types of training and how the skills would benefit PrEP delivery at an organizational‐ and system‐level. The same model practiced in New Zealand had incorporated the development of an online training module, with PrEP prescriber resources disseminated specifically to general practitioners in the management of PrEP, resulting an increase of PrEP providers from 0 to 25 in 10 cities in 2016 [46].

### 3.4 Construct 4: affordability

Affordability refers to the “economic capacity for people to spend resources and time to use appropriate service” [24]. PrEP is cost‐effective as proven by some modelling studies conducted in Germany [58], UK [59] and the Netherlands [60]. However, the high cost of PrEP remains the most commonly cited barrier globally [61]. While PrEP is generally not offered within the national healthcare system in many countries in the Asia‐Pacific, out‐of‐pocket payments could potentially make HIV prevention financially inaccessible to individuals with limited income. Sustainable funding is one of the major factors to support PrEP implementation in both high‐income countries and Low‐to‐Middle‐Income countries [62]. Under the typology of free‐based models either in public or community setting, PrEP medication and other testing services were delivered free for evidence‐based programmes with the funding support by pharmaceutical companies, international non‐profit organizations, international charitable groups, government, domestic research centres or donors. An array of research activities was carried out in the Asia‐Pacific for the purpose of identifying impacts in real‐world settings. Such activities did contribute resources in light of the scarcity of antiretrovirals in the region, albeit short‐term and lacking sustainability.

In contrast, the central tenant of the fee‐based public service model and fee‐based community setting model was the low affordable fee. The accessible price was guaranteed by the legal availability of generic formulations at one‐tenth or less of the price of the branded drug. PrEP‐30, the first affordable fee‐based and publicly available PrEP service in Thailand, offered PrEP at <US$1 (30 THB) per day, together with the provision of testings (HIV, hepatitis B and renal function testing), counselling, condoms and lubricants. The accumulated total number of users at about 2,000 by June 2018 accounted for more than one‐third of the all PrEP users in Thailand [29]. It was shown that MSM and other high‐risk individuals were willing to pay for PrEP if delivered at an affordable price [28, 29]. The increased uptake indicated that fee‐based PrEP at a modest price has gained ground and that has remained an important pathway for PrEP access even when free prescription of PrEP was available through other programmes [29]. In Vietnam, clients shared the cost by paying US$15 per month for participating in the KP‐led Prepped for PrEP‐P4P programme. Some 83% MSM and TGW were generally willing to pay [37].

In places where generic PrEP was not available, people with cost concern might have turned to self‐obtaining and self‐administering PrEP through online purchases from overseas internet pharmacies. Taiwan spearheaded the implementation of the PrEP‐PAPA Project to address the cost barrier. PrEP prescriptions were shipped to clients in Taiwan, following assessment made in a participating public hospital in Taiwan, where healthcare providers confirmed eligibility and electronically sent laboratory results to the collaborating private clinics in Thailand for verification and prescriptions. This project increased affordability and convenience by enabling 65 PrEP users to access PrEP by January 2018, with 46% of them formally enrolling in the CDC Demonstration Project [50]. As financial sustainability could determine access of PrEP in the long‐term, declining donor funds had become the major challenge faced by the KP‐led Prepped for PrEP‐P4P [37], and PrEP‐India project [40].

### 3.5 Construct 5: appropriateness

Healthcare accessibility requires appropriate level of services determined by “the fit between services and clients need, its timeliness, the amount of care spent in assessing health problems and determining the correct treatment…” [24]. Aligning to the principles of “needs‐based, demand‐driven, and client‐centered,” the Princess PrEP Programme under the free community setting model recruited CHW for the benefits of engaging individuals who “truly understands KP’s lifestyle” [35]. Not only were there peers to promote PrEP access by providing risk assessment for potential clients, clients’ decision in choosing PrEP remained autonomous without interference. Overall, this programme was assessed as an effective “de‐medicalization strategy” in which 52% users successfully accessed PrEP through this modality, as of June 2018 [35]. For clients in India, the PrEP‐India project was committed to appropriate service by applying individualized PrEP dispensation plan, so that clients could receive medication by weekly pick up at CBOs‐clinics or home delivery community mobilizers according to their needs [38, 40].

Assuring timely access and follow‐up is central to PrEP delivery. Timely access was not on the strategic agenda of any specific model, but relevant initiatives were identified. Rapid HIV testings were available in most of the programmes, but the details were not reported in some cases (Table 3). To further reduce access delay, PrEP prescription was available on the first appointment day for someone joining PrEP‐30 in Thailand [28, 29] and EPIC‐NSW in Australia at circumstances of normal renal function and HIV‐negative status [41]. The Princess PrEP Programme adopted “same‐day PrEP flow” as one strategy, amidst all strategies to maximize PrEP access in a simple and speedy way [34]. HIV‐negative clients enrolling in this programme could start same‐day PrEP, as dispensed by the CHW under doctor’s standing order [36].

### 3.6 Model preferences in the Asia‐Pacific

Of the four models characterized, the free community setting model was most commonly adopted in the Asia‐Pacific (Table 2). The strength of this model was its boost to the capacity of facility and human resources, utilizing CBOs‐operated clinics and community members as key resources, with enhanced “approachability”, “acceptability” and “appropriateness” (Figure 3). Similar model has also been implemented in Malaysia, where PrEP was delivered through a demonstration project in the community setting comprising clinics operated by private, CBO and academia [63]. Another strength was the focus on technology that had a positive impact on stigma‐free access of PrEP. Alongside the need for raising awareness, supportive environments and ensuring timely access were means for enabling the development of a sustainable PrEP programme. Despite the lower level of involvement of the CBOs in the free public service model adopted in Australia and New Zealand, big progresses have been made in improving “approachability,” “availability” and “appropriateness,” with attention paid to equity in accessing PrEP (Figure 3). While paying for PrEP at high cost was a practical concern, a common criticism of the fee‐based models was the challenge to stay competitive in attracting users who may otherwise prefer services belonging to the free‐based models. Here, the use of low‐cost generic PrEP showed significant cost‐saving, leading to an increased access of vulnerable populations. While this model has contributed to narrowing the gaps in PrEP accessibility, the availability of generic antiretrovirals was limited to some countries only and that their quality would need to be assured. Long‐term affordability of free‐based models, on the other hand, would need to be maximized to increase accessibility. Clearly, expanded access will not succeed without the commitment from the government through supporting policies and national financing. Recently, only a few countries have been able to provide comprehensive support, as in Australia which subsidized PrEP through the Pharmaceutical Benefits Scheme (PBS) as of 1 April 2018, and New Zealand where ongoing funding renewals were made for those meeting funding criteria as of 1 March 2018. The PBS increased affordability when PrEP users paid a small proportion (i.e. “co‐payment”) of the cost of the dispensed medication and the government subsidized the remainder. Funding has become more crucial for PrEP rollout. In Thailand, PrEP has recently been included under its Universal Health Coverage Scheme (from October 2019 onwards). Free PrEP was also being scale‐up in Vietnam in 26 provinces through PEPFAR, Global Fund and the government [64]. While PrEP remained relatively unaffordable in Taiwan, an update of the guidelines of 2018 specifying who would benefit most from PrEP was shown to have expedited programme implementation [65]. In developing countries with limited resources, sustainable national pharmaceutical budget planning to make PrEP affordable is essential. Where stigma and punitive laws directed towards key‐population are present in certain settings, the creation of conducive policy environment supporting PrEP affordability could alleviate barriers.

## 4 Conclusion

Our review has assessed the strengths and weaknesses of PrEP delivery activities across cities/countries in the Asia‐Pacific through the framework of the five constructs of healthcare accessibility. Our analysis highlighted characteristics of programmes with similarities and differences in their respective approaches to address issues relating to the accessibility of PrEP as a healthcare commodity and with regard to their public health impacts. The models identified in the Asia‐Pacific were derived from programmes in Australia, New Zealand, India, Philippines, Taiwan, Thailand and Vietnam. They were disseminated at varying scales and stages, some of which being scales‐ups, with a strong collaborative effort from various stakeholders to meet the needs of clients, provider and system‐level challenges. Our analyses suggested that evaluation of PrEP programmes based on the five constructs of healthcare accessibility can be more useful than conventional comparisons in regard to uptake alone. The framework serves as a valuable analytical tool to guide service design decisions for maximizing the short, medium and long‐term outcomes of PrEP. The impacts of these outcomes are in turn measurable by these constructs. We conclude that it contributes to bridging knowledge gaps between PrEP delivery and facilitators leading to accessibility (Figure 3).

We retrieved all relevant peer‐reviewed articles, conference papers and abstracts from an array of journals, and academic conferences including both quantitative and qualitative studies. The approach of data analysis – stemming from the use of spider charts – makes it effective in viewing and assessing the accessibility of PrEP multi‐dimensionally. However, issues in regard to scaling arise, in particular, when studies with different variables are grouped for comparison [66]. The dearth of availability of data of the ongoing and recently completed projects has limited our ability to make effective comparisons between different models. Outcome data for many of these delivery models are often limited in the published literature, but more accessible in recent conference presentations. Nevertheless, this review has provided an up‐to‐date assessment of the PrEP service models available in the Asia‐Pacific. While our review shows a predominance of quantitative studies with large sample sizes and numerical data for supporting generalization, studies involving a combination with qualitative approach are particularly useful to better understand the complex process of community engagement, facilitators of effective models and structural barriers for implementation plan. We are not proposing to identify a unified one‐size‐fits‐all model. We see the need to develop a model that fits the unique social and cultural background of HIV and its prevention, which is dependent on not just the epidemiological context but and the needs of the community. As PrEP delivery services continue to evolve, the application of the five constructs on improve accessibility could benchmark best practices, which would in turn allow more effective models to be designed in future.
